# The CoughRetrain Program: Restoring Control Through a Non-pharmacological Intervention

**DOI:** 10.1007/s00408-026-00899-7

**Published:** 2026-05-28

**Authors:** Ana Oliveira, Shirley Quach, Anastasia N.L. Newman, Renata Mancopes, Mustafaa Wahab, Elena Kum, Kim Smith, Dina Brooks, Imran Satia

**Affiliations:** 1 Respiratory Research and Rehabilitation Laboratory (Lab3R), School of Health Sciences (ESSUA), University of Aveiro, Aveiro, Portugal; 2 Department of Medical Sciences, iBiMED –Institute of Biomedicine, University of Aveiro, Aveiro, Portugal; 3 Lung Health Foundation, Toronto, Canada; 4 School of Rehabilitation Science, Faculty of Health Sciences, McMaster University, Hamilton, Canada; 5 Swallowing Rehabilitation Research Lab, KITE - University Health Network, Toronto, Canada; 6 Department of Medicine, McMaster University, Hamilton, ON Canada; 7 Department of Health Research Methods, Evidence, and Impact, McMaster University, Hamilton, ON Canada; 8 Firestone Institute for Respiratory Health, St. Joseph’s Healthcare, Hamilton, ON Canada

**Keywords:** Cough hypersensitivity syndrome, Self-efficacy, Motivational interviewing, Tele-rehabilitation

## Abstract

**Purpose:**

Non-pharmacological cough interventions are recommended for refractory chronic cough (RCC), yet structured development processes and integration of behavioral theory are rarely described. We report on the development and feasibility of CoughRetrain, a theory-informed telehealth behavioral program that integrates behavior-change strategies to support voluntary control of coughing in RCC.

**Methods:**

CoughRetrain was developed following Medical Research Council guidance through staged problem identification, evidence review, multidisciplinary refinement, remote adaptation, and formative testing in interstitial lung disease. The final five-session individual program integrates education, graded suppression practice, motivational interviewing, and goal setting. A single-group feasibility pilot in adults with RCC assessed adherence, fidelity, adverse events, general self-efficacy (New General Self-Efficacy Scale), cough-specific self-efficacy (Cough-Specific Self-Efficacy Scale), cough-related quality of life [Leicester Cough Questionnaire (LCQ)], cough severity visual analogue scale (CS-VAS), and 24-hour objective cough frequency.

**Results:**

Twelve participants were enrolled, and eleven (median age 51 [38–63] years; 55% female; median cough duration 6 [3–20] years) completed the program. All core components were delivered. Two non-serious adverse events occurred (urticaria; headache). General self-efficacy remained stable (mean change 0.04 ± 0.19). Cough-specific self-efficacy increased by 0.3 ± 0.6. LCQ increased by 3.6 ± 3.0 points (95%CI 1.5 to 5.6). CS-VAS decreased by 18.8 ± 25.0 mm (95%CI −35.7 to −1.9), and 24-hour cough frequency decreased by 48% (95%CI −74% to +3%).

**Conclusions:**

CoughRetrain is a safe and feasible telehealth intervention for RCC developed through a transparent, patient-informed process. Preliminary findings align with behavioral cough literature. Larger controlled studies evaluating long-term efficacy are warranted.

## Introduction

Chronic cough (CC), defined as a cough lasting 8 or more weeks [1], affects approximately 10% of adults worldwide and is among the most common reasons for primary care consultation [2]. One-third of cases are classified as refractory or unexplained (RCC/UCC), in which symptoms persist despite guideline-based management [3]. Individuals report daily coughing bouts, sleep disruption, fatigue, embarrassment, and social withdrawal, often accompanied by anxiety and depressive symptoms [4, 5]. The healthcare and societal impact of RCC is substantial, driven by repeated consultations, prolonged diagnostic pathways, and ineffective treatment cycles [6–8]. 

The persistence of cough despite guideline-based management has prompted reconsideration of the mechanisms underlying RCC. Neurophysiological, neuroimaging, and pharmacological evidence supports reframing RCC as a disease of neuronal dysregulation rather than an ongoing irritation of the airways or gastrointestinal tract [9]. The concept of cough hypersensitivity syndrome describes RCC as a condition driven by heightened sensitivity within peripheral sensory afferents and central neural networks [10]. Peripheral sensitization of vagal afferents increases neuronal responsiveness to thermal, mechanical and chemical stimuli, while central sensitization and impaired cortical inhibitory signaling further contribute to excessive coughing [11]. Neuroimaging and experimental studies with inhaled capsaicin demonstrate reduced activation of cortical inhibitory pathways that normally suppress coughing , contributing to a mismatch between the urge-to-cough and voluntary control [13]. In this context, repeated coughing may reinforce cough behavior over time [14]. 

This pathophysiology mirrors other chronic hypersensitivity conditions, particularly chronic pain, where persistent neuronal sensitization, impaired descending inhibition, and behavioral reinforcement maintain symptoms beyond the original trigger [15]. Accordingly, a biopsychosocial perspective, consistent with contemporary guideline-based pain management approaches [16, 17], may offer a more comprehensive framework for RCC. As in chronic pain rehabilitation, long-term improvement likely depends not only on reducing neuronal excitability but also on modifying learned response patterns and enhancing self-regulatory and inhibitory processes to shape responses to airway sensations [14]. 

Nevertheless, current treatments for RCC predominantly target neural excitability, while behavioral mechanisms of cough regulation remain less explicitly addressed [18]. Pharmacological agents (e.g., morphine, pregabalin, gabapentin, amitriptyline) may provide partial relief, but are limited by inconsistent effectiveness, adverse effects, accessibility, and adherence [19]. Importantly, these medications are not designed to target behavioral processes that perpetuate cough. Non-pharmacological interventions improve cough frequency and quality of life [14, 20], and typically emphasize suppression strategies and laryngeal techniques, with less explicit specification of behavioral mechanisms, such as self-efficacy and goal-directed regulation.

This gap creates an opportunity to further develop existing non-pharmacological approaches by explicitly integrating behavior-change mechanisms into intervention design. CoughRetrain is focused on education and behavioral changes that may influence sensory hypersensitivity, motor control, and cognitive–behavioral regulation to promote self-regulation and restoration of voluntary control over the cough reflex. This paper describes its theoretical rationale and development and presents preliminary feasibility data in individuals with RCC.

## Methods

**Fig. 1:**
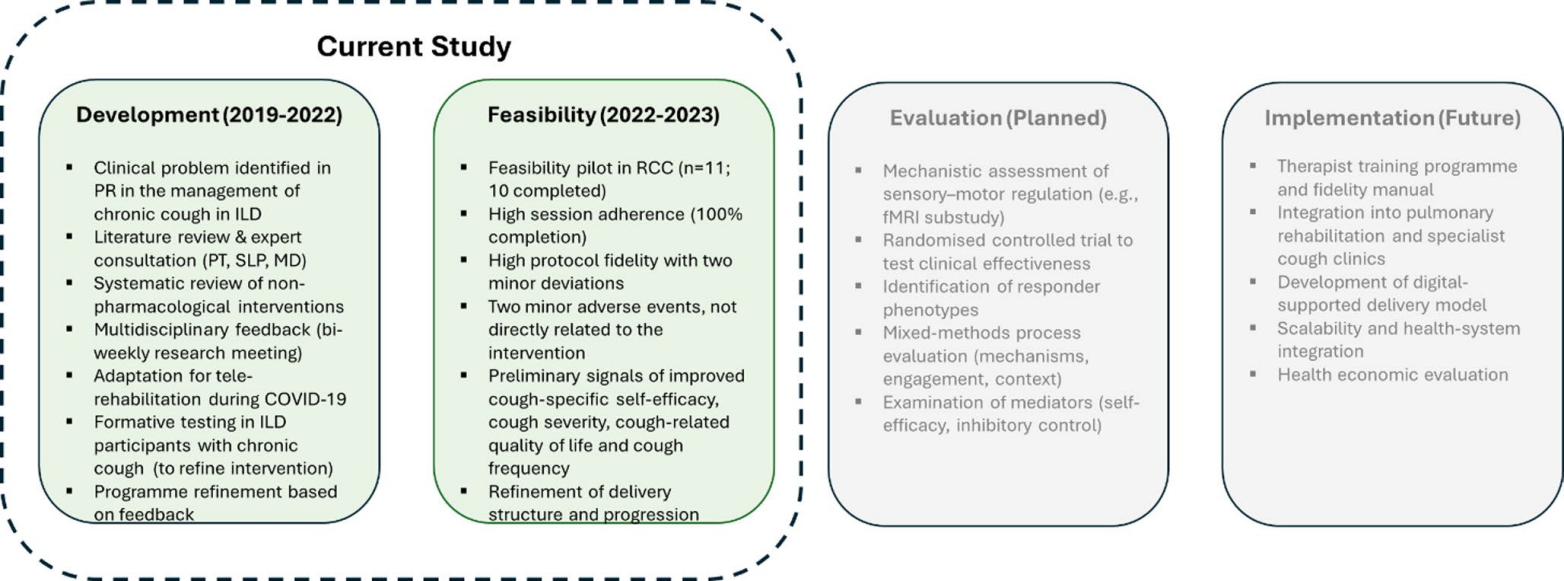
Development and feasibility testing of the CoughRetain program within the Medical Research Council framework. fMRI, functional magnetic resonance imaging; ILD, interstitial lung disease; MD, medical doctor; PR, pulmonary rehabilitation; PT, physiotherapist; RCC, refractory chronic cough; SLP, speech language pathologist

The development of CoughRetrain followed an iterative, evidence-informed process consistent with the principles in the Medical Research Council (MRC) framework for developing and evaluating complex interventions [21] (Fig. 1). The process combined problem identification, evidence synthesis, expert and stakeholder consultation, and feasibility considerations. Behavioral components were informed by the COM-B model within the Behavior Change Wheel [22], which was used to identify key determinants of cough behaviour (capability, opportunity, and motivation) and guide the selection of intervention strategies. Education and skills training were incorporated to enhance psychological and physical capability, structured sessions and supervised practice to create opportunities for behavioural rehearsal, and goal setting, graded exposure, and motivational interviewing to support both reflective and automatic motivation. These components were operationalised within a predefined session structure combining education, practice, feedback, and goal setting. The final program was documented using the TIDieR checklist [23] to enhance transparency and replicability. A summary of program evolution is provided in Table S1.

### Development Phase

#### Phase 1 – Identification of the Clinical Problem

In July 2019, informal clinical discussions with physiotherapists at the Pulmonary Rehabilitation Program of West Park Healthcare Centre (Toronto, Canada) identified difficulties managing patients with interstitial lung disease who experienced persistent coughing that limited exercise participation, highlighting an unmet need for structured behavioral approaches to cough management within rehabilitation settings.

#### Phase 2 – Evidence Review and Expert Consultation

A focused review of the literature on RCC and non-pharmacological interventions revealed that existing programs were typically delivered by rehabilitation health professionals [24]. While these interventions outlined the main components of the therapies and provide a strong foundation for clinicians, they often lacked a detailed description of therapeutic content, theoretical underpinnings, or explicit behavior-change strategies aimed at enhancing self-efficacy and supporting goal-directed practice. International experts in cough control therapy were consulted [25, 26] to understand their clinical frameworks and implementation strategies, as well as expert physicians on RCC physiology [27], to align behavioral principles with current understanding of cough neurophysiology and pharmacological management.

#### Phase 3 – Evidence Synthesis

**Fig. 2:**
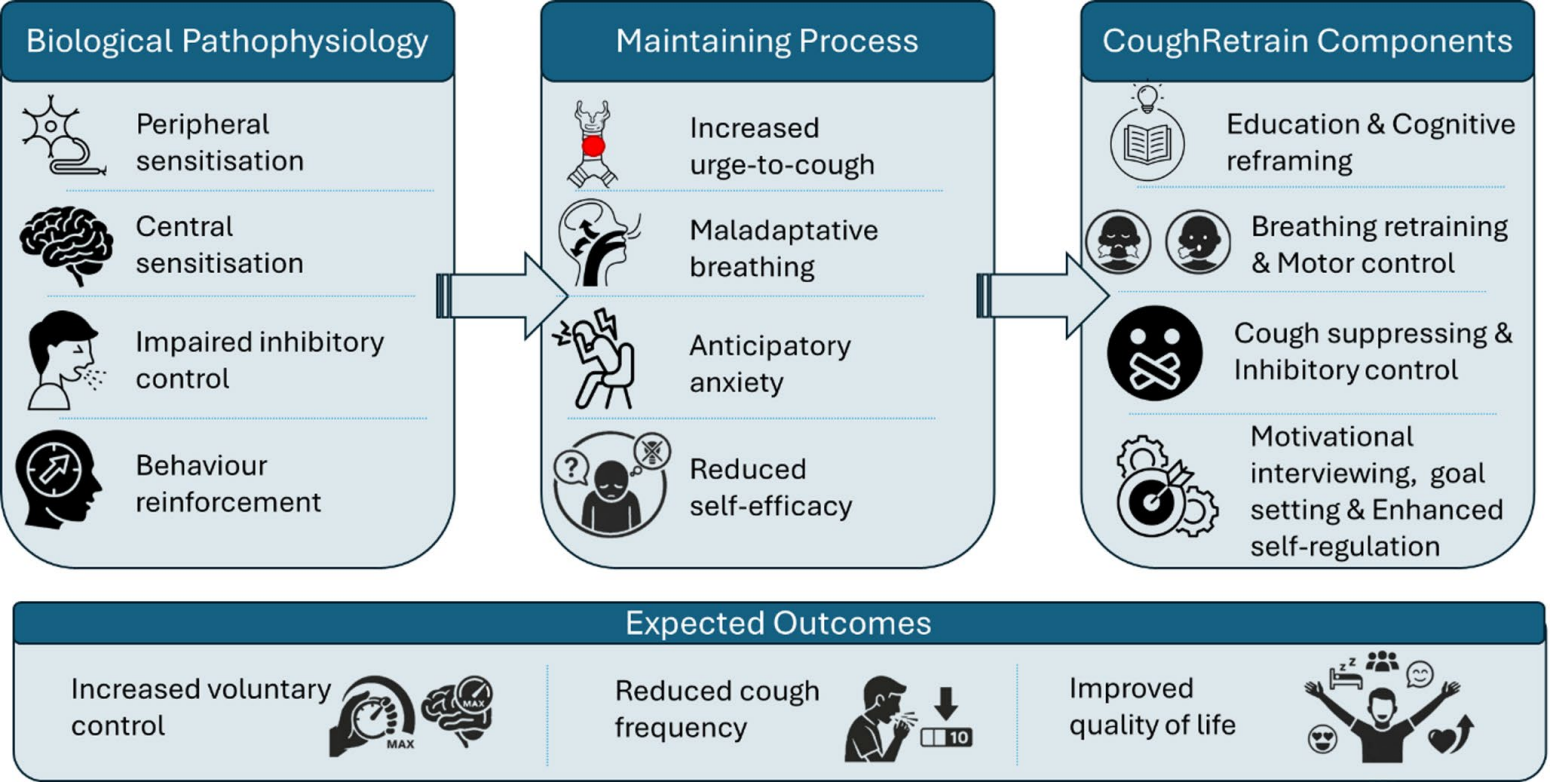
Conceptual framework for the CoughRetrain program in refractory chronic cough

A systematic review was subsequently undertaken to examine the effects of non-pharmacological interventions for individuals with chronic respiratory diseases and CC [28]. The findings consistently indicated that multimodal interventions, combining education, breathing and laryngeal retraining, and cough-suppression practice produced greater improvements in cough frequency and quality of life than single-component approaches. These results informed the conceptual and structural framework of the CoughRetrain program (Fig. 2).

#### Phase 4 – Multidisciplinary Refinement

The draft program was presented during a multidisciplinary research meeting by the West Park Healthcare Centre Research Department in December 2019. Feedback focused on conceptual framing and session sequencing, leading to redistribution of educational content, introduction of a predefined session structure, and alignment of educational topics with therapeutic techniques (see Table S1 for full detail).

#### Phase 5 – Adaptation for Virtual Delivery

Originally designed for in-person delivery, CoughRetrain was adapted for tele-rehabilitation in 2020 due to COVID-19 restrictions. Materials and therapist guidance were revised to support supervised practice, interactive education, and goal setting via secure videoconferencing.

#### Phase 6 – Formative Testing and Refinement

Formative testing (Clinicaltrials.gov: NCT04767074; Ethics: ref. 20-005-WP, Hamilton Integrated Research Ethics Boards (HiREB) #13017) was explored in 2021–2023 with 11 individuals with interstitial lung disease and CC enrolled in the pulmonary rehabilitation program at West Park Healthcare Centre. A full diagnostic work-up to classify RCC was not undertaken.

The initial program comprised four sessions over the final two weeks of pulmonary rehabilitation (two sessions/week). Eight individuals completed the program and participated in semi-structured interviews exploring perceptions of program content, structure, and usefulness. Interviews were audio-recorded, transcribed, and analysed using inductive thematic analysis [29] to identify key areas for improvement. Four main themes emerged:

1. Developing control through knowledge and practice.
2. Structure and pacing to support learning and consolidation.
3. Clarity and integration of content.
4. Feasibility and acceptability of telehealth delivery.

Participants described developing awareness of their cough and learning practical techniques that enhanced their sense of control and confidence. They valued the educational content and accessibility via telehealth and suggested extending the interval between sessions to allow more practice, adding a review session for feedback and reinforcement, and clarifying the speech-language content with simpler explanations and visual demonstrations. These insights directly informed the final five-week structure of CoughRetrain and the adaptation of its materials for telehealth delivery (Table S2).

### Feasibility Phase in Refractory Chronic Cough

Feasibility of telehealth delivery was evaluated as a secondary analysis of the PROCOUGH study (HiREB #11231), a prospective, single-centre, investigator-initiated observational cohort study conducted at McMaster University Medical Centre (2021–2025). Participants completed baseline and post-intervention assessments of objective and subjective cough (6–8 weeks). This phase assessed acceptability, practicality, safety, and preliminary effects associated with the intervention. All participants provided written informed consent.

#### Participants and Recruitment

Adults aged 18–80 years referred for specialist evaluation of CC were eligible. Inclusion required a normal chest radiograph and no airflow obstruction (FEV_1_/FVC ≥ 0.70). Exclusion criteria included current smoking or smoking within the previous 6 months with a cumulative history of ≥ 20 pack-years, active respiratory infection or asthma exacerbation within 4 weeks prior to enrolment, and significant underlying pulmonary disease. Use of centrally acting neuromodulators required discontinuation prior to enrolment where appropriate. Full details on participants’ recruitment are described elsewhere [30]. 

#### Intervention Delivery

A treatment algorithm based on the European Respiratory Society guidelines [1] and additional investigations (e.g., methacholine challenge, FeNO, sputum differential cell count) was used to identify individuals with RCC. Participants with persistent cough following initial diagnostic work-up, consistent with RCC/UCC, were offered CoughRetrain. Full details on the treatment algorithm are provided elsewhere [30]. 

CoughRetrain was delivered as a five-session individual intervention over six weeks via videoconferencing (Table 1). Sessions (45–60 min) were delivered by a physiotherapist and a speech language therapist trained in RCC management and motivational interviewing, using a standardised manual. The program combined education, breathing retraining, laryngeal modulation (taught by a speech language therapist in session 3), cough suppression training, and behavioral strategies to support adherence and self-regulation [25, 26]. Sessions followed a consistent structure including review of home practice, targeted education, supervised practice, and collaborative goal setting.

**Table 1:** Structured delivery of the CoughRetrain program

Session/Week	Educational component	Clinical skills & techniques	Motivational interview prompts	Action planning & monitoring
1 Week 1	• Cough neurophysiology • Reflexive (brainstem) vs. voluntary (cortical) control • Trigger awareness	• Identify early urge-to-cough • Introduce suppression (dry swallow ± controlled breathing) • Trigger identification (“mapping” of timing/context)	• “What do you understand about why your cough persists?” – Personalize information to participant’s knowledge • Confidence scale (0–10)	• Define one predictable context for inhibition practice
2 Week 2	• Sensitization cycle • Irritation–cough loop • Rationale for strengthening inhibition • Dry vs. productive cough (if applicable)	• Reinforce suppression • Hydration optimization	• “What happened when you tried to suppress your cough?” – Reinforce the positive aspects • Confidence scale (0–10)	• Apply techniques during predictable triggers • Adjust weekly goals & cough suppression techniques based on patient feedback
3 Week 3	• Vocal folds & laryngeal hygiene	• Laryngeal hygiene & relaxation • Breathing and airway clearance techniques (if applicable) • Technique integration	• “What was the most cough-related challenging situation you had during last week? How did you manage it?” • Confidence scale (0–10)	• Collaborative planning of graded exposure tasks • Problem-solving for inhibitory breakdowns
4 Week 4	• Neural regulation consolidation • Repetition strengthens cortical control	• Flexible strategy integration • Develop emergency suppression plan	• “How confident are you that you can delay a cough unexpectedly?” • “What evidence do you have that you can control it?” • Confidence scale (0–10)	• Practice in high-trigger contexts • Refine emergency plan
5 Week 6	• Relapse prevention • Maintenance of inhibitory capacity	• Optimize preferred strategies • Independent implementation	• “When your cough reappears, what is the first action you can take to regain control?” • “How will you remind yourself that you’ve succeeded before?” • Confidence scale (0–10)	• Final maintenance plan • Identify & role play long-term high-risk situations

Participants were instructed to practice strategies daily between sessions. Practice tasks were collaboratively defined and graded according to individual cough triggers, progressing from low-trigger to more challenging situations. Motivational interviewing techniques were embedded within goal setting and skill practice to address ambivalence, reinforce autonomy, and support adherence to suppression and exposure tasks [31]. 

Further details are provided in Supplementary Material 1.

#### Feasibility and Preliminary Clinical Outcomes

Feasibility was evaluated through session adherence, adverse events, and treatment fidelity. Session adherence was defined as attendance at scheduled sessions and summarized as the proportion of participants completing all sessions and the mean number of sessions attended. Adverse events were monitored at each session through structured enquiry and spontaneous reporting and classified as related or unrelated to the intervention.

Treatment fidelity was assessed using a structured healthcare provider checklist completed after each session [32, 33]. The checklist captured delivery of core components (education, cough suppression training, breathing retraining, laryngeal strategies, and collaborative goal setting) and behavioral process elements reflecting integration of motivational interviewing techniques (Table S3). Fidelity was summarized as the proportion of required components delivered per session and averaged across participants to estimate protocol adherence.

Preliminary clinical outcomes were explored to characterize potential signals of change and inform future trial design. Self-efficacy was assessed using the New General Self-Efficacy Scale [34] (NGSE; 8 items; 0–5, with higher scores indicating greater general self-efficacy), which has demonstrated construct validity and internal consistency, and a cough-specific self-efficacy scale developed according to Bandura’s framework for task-specific self-efficacy assessment [35] (5 items; 0–5, with higher scores indicating greater cough-related self-efficacy). Cough severity was assessed using the Cough Severity Visual Analogue Scale (CS-VAS; 0–100 mm, from “no cough” to “worst cough”; higher scores indicate greater cough severity), which has demonstrated convergent and known-groups validity and responsiveness in individuals with chronic cough [36]. Cough-related quality of life was assessed using the Leicester Cough Questionnaire (LCQ; 19 items across physical, psychological, and social domains; total score range 3–21, with higher scores indicating better cough-related quality of life), which has demonstrated validity, repeatability, internal consistency, and responsiveness in chronic cough [37]. Objective cough frequency was assessed using 24-hour ambulatory cough monitoring with the VitaloJAK system, a semi-automated cough monitoring system validated for objective measurement of cough frequency in ambulatory recordings [38]. 

Outcomes were summarized descriptively at baseline and post-intervention. Continuous variables are presented as mean±standard deviation. Geometric means were used to account for skewed cough frequency data. They reflect central tendency after log-transformation and are obtained by back-transforming the mean of the log values.

Mean change scores are presented with 95% confidence intervals. Given the exploratory design and small sample size, analyses focused on variability and individual response patterns rather than formal hypothesis testing. The number of participants achieving the Minimal Important Difference (MID) for the CS-VAS (≥ 30 mm) [36], LCQ (≥ 1.3) [39] and objective cough frequency (≥ 30% change) [40] was recorded.

## Results

Of 100 participants enrolled in the PROCOUGH cohort, 12 participants who met predefined RCC criteria according to the study treatment algorithm were offered the CoughRetrain program. One withdrew prior to initiation due to time constraints. Descriptive characteristics of the 11 participants who completed the program are presented in Table 2.

**Table 2:** Baseline characteristics of participants completing the CoughRetrain program

	Sex	Age	BMI	Smoking status	Cough duration (years)	LCQ (total score)	CS-VAS (mm)	24 h cough counts (coughs/hr)
Pt 1	Female	51	30.9	Never	20	14.1	32	62.0
Pt 2	Male	60	28.4	Never	5.5	17.1	24	6.4
Pt 3	Male	38	25.1	Never	9	15.5	29	25.1
Pt 4	Female	37	24.0	Ex-smoker	3	10.4	92	5.2
Pt 5	Male	18	35.5	Never	1	5.5	79	229.5
Pt 6	Female	67	25.2	Ex-smoker	30	4.8	96	39.9
Pt 7	Female	71	22.8	Never	22	8.1	75	33.4
Pt 8	Female	65	29.0	Ex-smoker	6	15.4	41	7.5
Pt 9	Male	20	20.8	Never	3.5	10.4	75	10.2
Pt 10	Female	41	20.5	Never	1	11.6	41	13.4
Pt 11	Female	54	31.5	Ex-smoker	10	8.0	79	18.5
Summary	55% females	Median 51 (Q1 38; Q3 63)	Median 25 (Q1 23; Q3 30)	63% never smoked	Median 6 (Q1 3; Q3 20)	Mean 11.0 (SD 4.2)	Mean 60.3 (SD 26.9)	Geo. mean 20.7 (SD 3.09)

### Feasibility Outcomes

All participants who initiated the program completed all five sessions (100%; mean sessions attended 5.0). Sessions were delivered within the planned timeframe, and all predefined core components were implemented across participants. Two minor protocol deviations occurred: delayed introduction of suppression techniques in one participant and independent completion of written goal setting outside the session in another.

Two adverse events were reported (urticaria, *n* = 1; headache, *n* = 1), with both classified as unrelated to the intervention. No serious adverse events occurred.

### Preliminary Clinical Outcomes

**Fig. 3:**
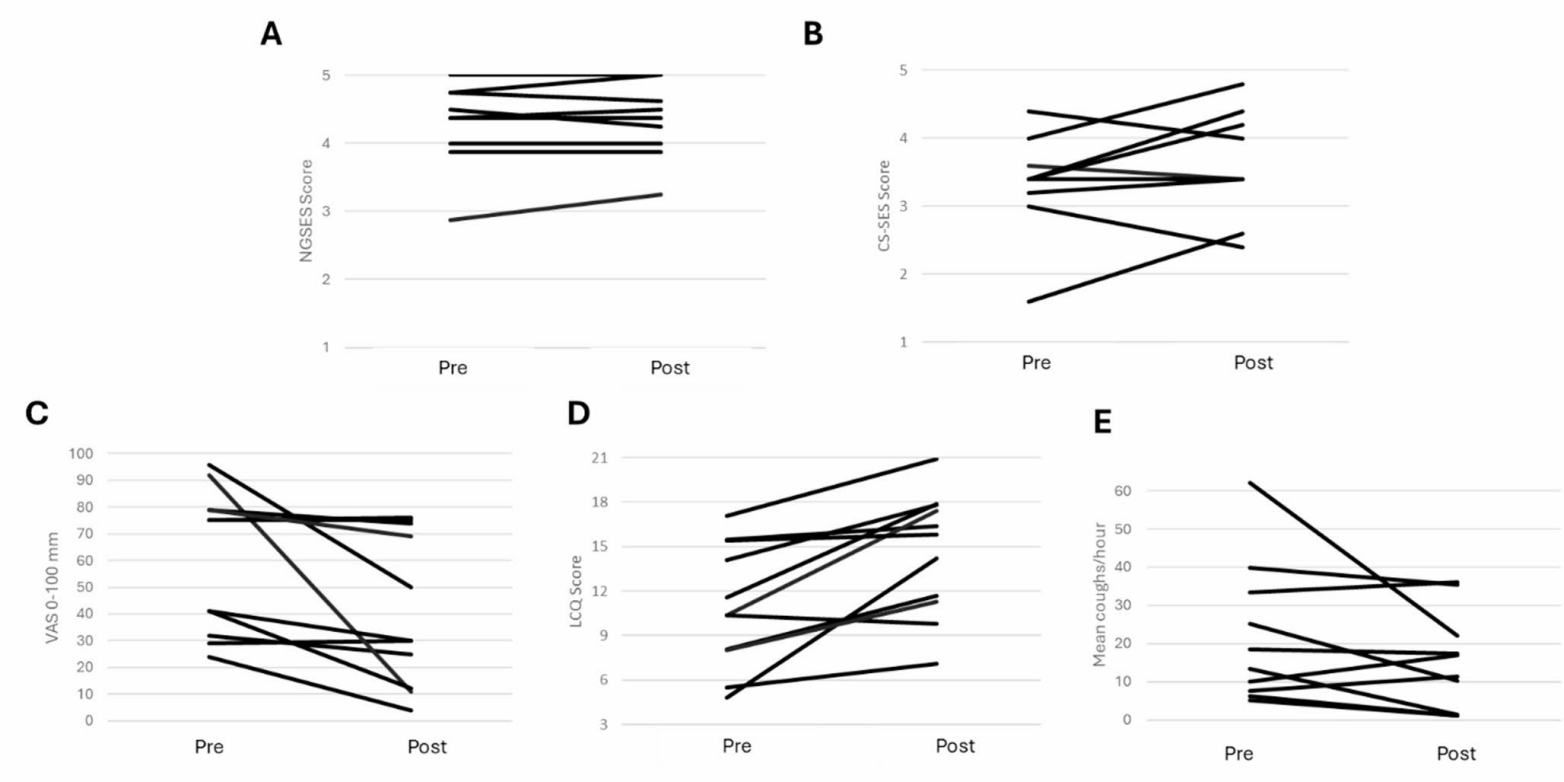
- Individual changes in outcomes following CoughRetrain. Plots showing individual participant trajectories from baseline (Pre) to post-intervention (Post): **A** New General Self-Efficacy Scale (NGSES; 0–5); **B** Cough-Specific Self-Efficacy Scale (CS-SES; 0–5); **C** Cough Severity Visual Analogue Scale (VAS, 0–100); **D** Leicester Cough Questionnaire (LCQ, 3–21); **E** 24-hour objective cough frequency (mean coughs/hour). Higher scores indicate improvement for NGSES, cough-specific self-efficacy and LCQ; lower scores indicate improvement for VAS and cough frequency

Preliminary clinical outcomes are in Fig. 3 and in Supplementary Table S4. Two participants did not complete post-intervention NGSES and CS-SES assessments, and one did not complete objective cough monitoring. General self-efficacy increased by 0.04 ± 0.19 (95% CI − 0.11 to 0.19), with 7/9 participants unchanged. Cough-specific self-efficacy increased by 0.3 ± 0.6; 95% CI −0.16 to 0.76 (6/9 participants improving). LCQ scores increased from 11.0 ± 4.2 to 14.6 ± 4.1 (mean change of + 3.6 ± 3.0 (95% CI 1.5 to 5.6), with 8/11 participants (72%) exceeding the MID. Cough severity (VAS) decreased from 60.3 ± 26.9 to 41.5 ± 28.2 mm (mean change −18.8 ± 25.0; 95% CI −35.7 to −1.9), with 3/11 (27%) exceeding the MID. Objective 24-hour cough frequency decreased from a geometric mean of 16.3 coughs/hour (GSD 2.3) to 8.4 coughs/hour (GSD 4.0), corresponding to a 48% decrease (95% CI −74% to +3%). Five of 10 participants (50%) achieved the ≥ 30% MID.

## Discussion

This study describes the iterative development and preliminary feasibility of CoughRetrain, a mechanism-informed behavioral program for RCC. The final telehealth program demonstrated high adherence, strong protocol fidelity, and no intervention-related adverse events. Preliminary data suggest potential improvements in cough-specific self-efficacy and quality of life, while general self-efficacy remained unchanged, consistent with the program’s targeted focus on cough-specific inhibitory control.

Similar to in-person cough control therapy, recent online interventions show that core components of cough management (i.e., education, suppression techniques, laryngeal hygiene, psychosocial support) can be delivered remotely with clinically meaningful improvements in cough-related quality of life [41–45]. These interventions include asynchronous [42] and virtual group-based delivery models [41, 43, 45], which differ in their emphasis on accessibility, scalability and peer interaction. CoughRetrain builds on these approaches but differs in its structured behavioural progression, integrating graded exposure to triggers, confidence-based task progression, and motivational interviewing to support self-regulation. This structure provides clearer specification of behavioral mechanisms and a reproducible framework for telehealth delivery. Comparing these different approaches may help identify areas for optimization, particularly the relative contribution of delivery format (individual vs. group; synchronous vs. asynchronous), behavioral structure (e.g., graded trigger exposure, and confidence-based progression) to treatment engagement, self-efficacy, and sustained cough control. Elements of existing web-based and group cough programs may also inform future refinements of CoughRetrain, including hybrid models or peer interaction [41–45]. 

A distinguishing feature of CoughRetrain is its staged, patient-informed development prior to feasibility evaluation. Testing in individuals with interstitial lung disease and CC informed pacing, session spacing to consolidate techniques, and clearer behavioral sequencing, with stronger integration of goal setting. This iterative process ensured that the final program reflected alignment between theoretical framing and users’ needs.

Although this feasibility study was not designed to evaluate effectiveness, the magnitude and direction of changes observed were broadly consistent with previous behavioural cough interventions [20, 25, 41, 42, 44, 45]. Improvements in cough-related quality of life exceeded the LCQ minimal important difference, whereas changes in cough severity and objective cough frequency were more modest and variable. Similar patterns have been reported in previous behavioural cough studies, where patient-reported outcomes appeared to improve more consistently than objective cough frequency measures [20]. These comparisons should be interpreted cautiously given differences in study design, populations, intervention components, and the exploratory nature of this study.

A trend was more evident for cough-specific than general self-efficacy. While previous interventions have reported improvements in self-belief, they have not distinguished between domain-specific and global confidence [41]. CoughRetrain emphasis on graded suppression practice and context-specific inhibitory control may explain this trend. This pattern is consistent with contemporary models of RCC that conceptualize the condition as involving impaired central regulation of cough behavior [14, 46]. These findings support further evaluation of CoughRetrain in controlled trials to examine its effects on cough frequency and underlying behavioural mechanisms.

### Strengths & Limitations

The principal strength of this study lies in the structured, theory-informed and patient-refined development of the intervention, with clear documentation from formative testing to feasibility evaluation. Integration of behavioral theory, graded exposure to cough triggers, and motivational interviewing within a predefined session structure enhances conceptual clarity and reproducibility. Fidelity monitoring and objective cough measurement further strengthens methodological rigor. An additional strength is the applicability of the program across related clinical populations, from individuals with interstitial lung disease to those with RCC, supporting the adaptability of the behavioral framework across contexts of CC. Finally, CoughRetrain was designed to be deliverable by trained clinicians within multidisciplinary respiratory care settings, thereby supporting broader implementation.

Limitations include the small sample size and single-centre design, which limit generalizability and preclude inference regarding effectiveness. The program was initially refined in individuals with interstitial lung disease and CC. As this population was not restricted to RCC, aspects of intervention design may not fully reflect the needs of all RCC populations, despite feasibility being demonstrated in this cohort.

## Conclusion

CoughRetrain is a structured, theory-informed behavioral program for RCC developed through an iterative, patient-informed process. The program was feasible, safe, and showed clinically meaningful improvements consistent with existing behavioral cough interventions. Observed changes in cough-specific self-efficacy are consistent with the program’s focus on self-regulation. Although effectiveness cannot be established in this feasibility study, the program provides a reproducible framework for future controlled evaluation and integration into respiratory care.

## Data Availability

The data that support the findings of this study are available from the authors upon reasonable request.
